# A smart insole system capable of identifying proper heel raise posture for chronic ankle instability rehabilitation

**DOI:** 10.1038/s41598-022-14313-8

**Published:** 2022-06-24

**Authors:** Jaewook Kim, Seonghyun Kang, Seung-Jong Kim

**Affiliations:** Department of Biomedical Engineering, Korea University College of Medicine, Seoul, 02841 South Korea

**Keywords:** Biomedical engineering, Rehabilitation

## Abstract

Heel raise is widely prescribed to patients with chronic ankle instability in order to strengthen the Peroneus Longus muscle (PL) which supports the weakened lateral collateral ligaments. While the exercise itself is intuitive, ankle orientation is of particular importance because heel raises performed with inversion do not well recruit the PL. This implies that proper execution is imperative and a means to assess heel raise training sessions is needed. In this study we present a smart insole system capable of identifying heel raise events and its corresponding rise, hold and drop phases, which allows for a more descriptive analysis. The results from our heel raise sessions, which consist of four different variants performed by five healthy subjects, suggest that medial–lateral foot pressure distribution and foot orientation are needed to differentiate heel raises performed with ankle eversion and inversion. We go further and substantiate that proper execution, detected by our system, indeed leads to increased PL activation by analyzing the electromyography signals. We believe that the proposed system may provide clinicians with invaluable information regarding onsite as well as at-home training and possibly, with biofeedback, serve as foundation for software as a medical device.

## Introduction

The ankle–foot complex plays an integral role in maintaining balance and stability, because it is part of the body that directly interacts with the ground. Therefore, the ankle must be able to transiently absorb impact and transmit propulsive forces as well as serve as a static foundation^1–3^. This requires meticulous flexibility and rigidity control, facilitated by complex coordination of multiple lower leg muscles, such as the Tibialis Posterior, Tibialis Anterior, Peroneus Brevis, and Peroneus Longus (PL), Soleus (SL), Gastrocnemius Medialis and Lateralis (GM, GL)^4–6^.

However, it is reported that approximately a quarter of the population is afflicted with chronic ankle instability (CAI)^7–9^ which elicits impaired mobility, poor function and osteoarthritis^10–12^. The actual number is estimated to be much higher because it is often not formally diagnosed until the later stages when more prominent complications are elicited^13^. CAI is a condition where the ankle is prone to lateral ankle sprains and frequent “giving away” even during routine activities of daily living such as walking. It is widely accepted that CAI itself is a complication caused by incompletely healed lateral collateral ligaments from a previous ankle sprain injury^14–16^. Thus, it is imperative that proper treatment for CAI is facilitated until complete rehabilitation^16–18^.

Heel raise is a popular non-surgical approach to prevent and/or treat CAI by promoting muscle strength, joint stability, and balance control^19–21^. While apparently simple, this exercise is precarious because the muscles that are recruited and subsequently strengthened vastly differ upon foot position and eversion/inversion angles. More specifically, while plantar flexors such as SL, GM and GL are well activated irrelevant of ankle eversion/inversion angles, a significant increase in PL recruitment is reported with ankle eversion^4,22–25^. Thus, when heel raise is incorporated into a regiment for CAI rehabilitation, which aims to provide additional support for weakened lateral collateral ligament complex by strengthening the PL muscle, it is vital that ankle eversion is maintained throughout the exercise^26–28^. Failure to do so would not only be ineffective but may encourage ankle inversion patterns which could further exacerbate the preexisting condition.

In specialized environments, guidance by trained medical specialists can be utilized to help ensure proper heel raise form. However, frequent training is necessary for desirable rehabilitative outcomes, and it cannot be expected for each session to be facilitated under supervised environments^29,30^. Thus, simple yet effective interventions such as keeping a lacrosse ball from dropping in between the heels or placing a coin under the medial forefoot have been devised^19,31^. With copious wearable healthcare products available in the current market, it may be possible to quantitatively assess, track and further guide proper heel raise execution using a smart insole^32–34^.

In efforts to facilitate PL strengthening and assess proper posture, we propose a heel raise monitoring system utilizing an FDA approved smart insole developed by Salted ltd. which offers foot pressure distribution data via four force sensing resistor (FSR) sensors, and three axis acceleration data which can be used assess foot orientation during static conditions. This data was compared with Pedar-X insoles and Delsys Trigno sensors to ensure that it well represents how the heel raises were performed. An automatic heel raise segmentation method was developed to detect heel raise events and moreover the rise, hold and drop phases such that electromyography (EMG) signals could be analyzed according to their concentric, isometric, and eccentric muscle phases. Four variations of the heel raise exercise, previously reported to have significantly different PL recruitment levels, were performed via five healthy subjects to test our proposed system. We find that using medial partial pressure (MPP) and insole acceleration, our system can robustly differentiate heel raises performed with ankle eversion and inversion regardless of single leg or double leg conditions. Furthermore, proper posture, identified by our system, indeed corresponds with higher PL activation levels especially during the concentric rise phase.

## Methods

### Participants

Five healthy male subjects (age: 29.00 ± 5.15 years, height: 176.80 ± 4.14 cm, and weight: 87.00 ± 8.51 kg) that participated in this study had no prior history of lower limb injuries (previous 6 months). All participants provided a written informed consent and research ethics of human experiments was ensured by conducting the sessions in accordance with the contents approved by the Institutional Review Board of Korea University College of Medicine (IRB No. 2021-0120-01). All experiments were carried out in accordance with the approved guidelines and with the Declaration of Helsinki. The raw data supporting the findings of this study are available upon request to the corresponding author.

### Experimental procedures

**Figure 1:**
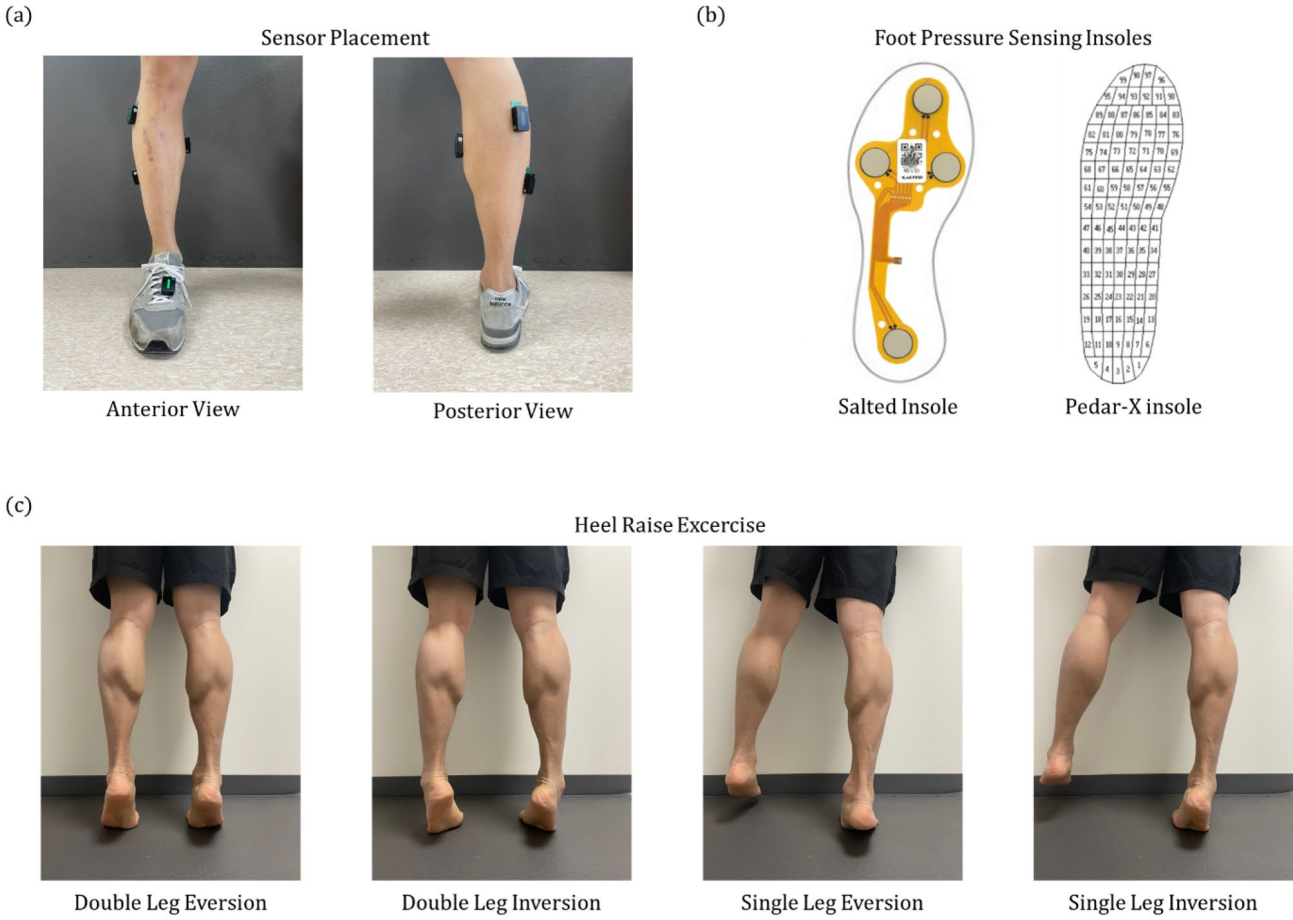
Experimental procedures for heel raise sessions. (**a**) Delsys Trigno sensor placements. The EMG sensors were placed on the PL, GM, GL and SL of the right leg. An additional sensor was placed on the shoe above the dorsal aspect of the 2nd tarso-metatarsal joint in order to quantitatively monitor the foot orientation during heel raise. (**b**) Two foot pressure insoles were used in this study. The sensor arrangements within the insoles are shown for Salted smart insoles and Pedar-X on left and right panels, respectively. (**c**) Heel raise variants. The four different heel raises that were performed DL-EV, DL-IV, SL-EV and SL-IV are shown from left to right.

In order to investigate whether the data acquired from the smart insoles are sufficient to differentiate heel raise performed with ankle inversion/eversion and furthermore elicit PL activation, we simultaneously measured foot orientation and muscle activity using IMU sensors and EMG sensors, respectively (Trigno™ wireless system, Delsys Inc., USA). The IMU sensor was placed above the dorsal aspect of the 2nd tarso-metatarsal joint and the EMG sensors were positioned on the PL, GM, GL and SL as shown in Fig. 1a. The participants were asked to raise the heels as high as possible while maintaining knee extension^19,21,22^. A total of five repetitions of the following heel raise variants: double leg with ankle eversion (DL-EV), double leg with ankle inversion (DL-IV), single leg with ankle eversion (SL-EV) and single leg with ankle inversion (SL-IV) were conducted (Fig. 1c). The heel raise sessions were monitored and cued by an orthopedic physician in order to ensure the exercise was performed correctly and that each heel raise was controlled to have an idle, rise, hold, and drop phase that lasted 5, 1, 3 and 1 sec, respectively.

### Signal processing

The smart insoles (Salted ltd., South Korea) provide wireless transmission of four channel foot pressure and three channel acceleration data at a sampling rate of 30 Hz. This fully mobile system, which requires no cables or separate battery packs, obtains foot pressure data via FSR sensors positioned beneath the toe, heel, 1st and 4th metatarsal head (Fig. 1b). Furthermore, a 3-axis accelerometer and a processor are embedded beneath the 2nd and 3rd metatarsal base. In order to validate the results obtained from this system, we also performed duplicates of all four heel raise variant using the Pedar-X system (Novel Inc., Germany) which utilizes 99 capacitive force sensors that cover entirety of the insole (Fig. 1b).

**Figure 2:**
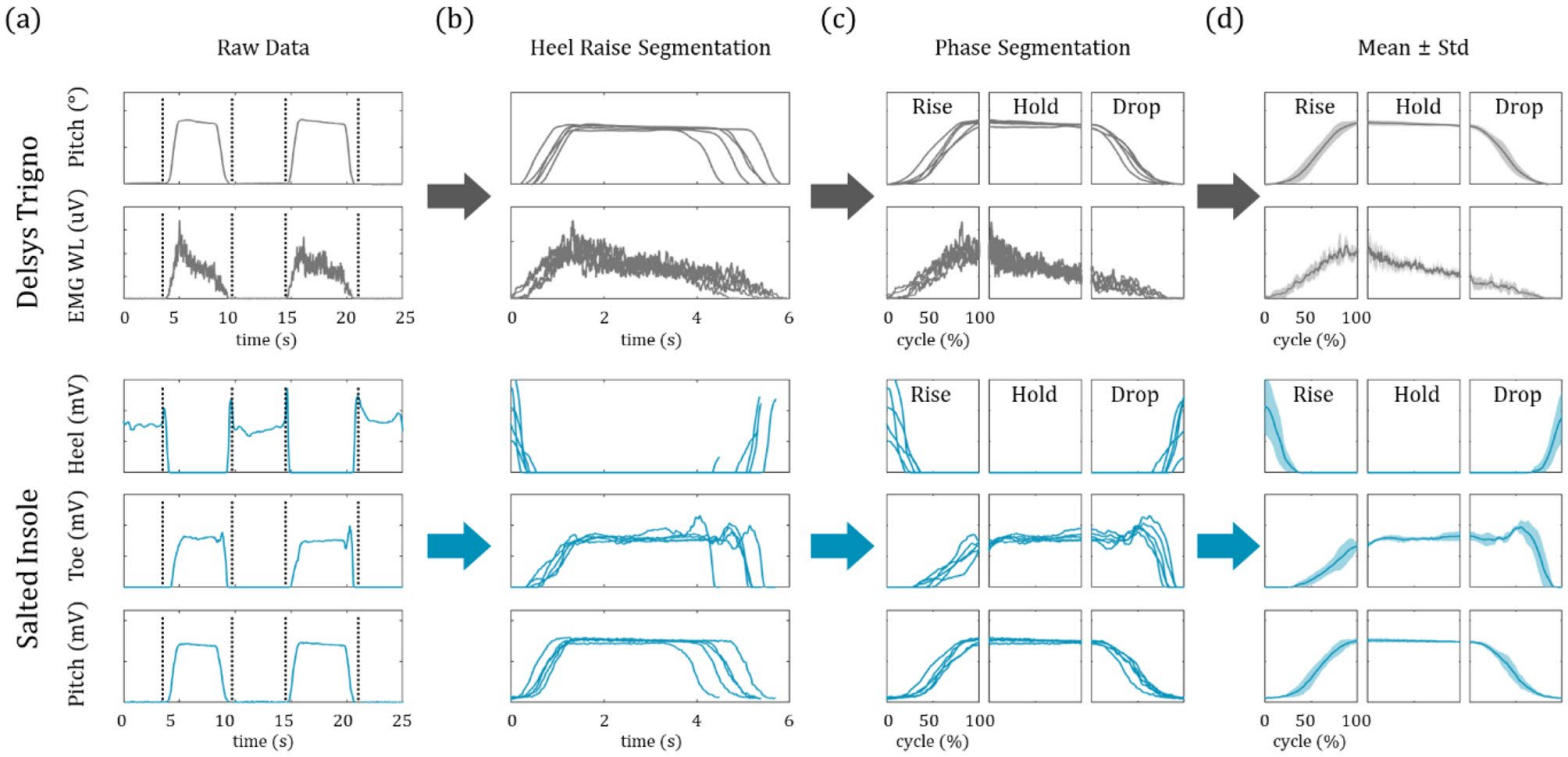
Automatic segmentation of heel raise events and data processing. (**a**) The data acquired from Delsys Trigno sensors and insoles are first synchronized. The initiation and termination of the heel raise events are determined by applying a simple threshold on foot pitch. (**b**) The synchronized signals are then collectively segmented and the rise, hold and drop phases are detected via pitch gradient. (**c**) The signals are further segmented into their respective phases and normalized along time axis such that they are represented in percentage of phase. (**d**) The mean and standard deviation are plotted against phase percentage as line and area, respectively.

Initially, the data acquired from the IMU, EMG sensors and insoles are synchronized and the EMG waveform length (WL) is calculated (Fig. 2a). Then, the foot pressure data and EMG WLs were segmented according to the heel raise trials and furthermore each phase by applying the simple aforementioned heel raise segmentation algorithm (Fig. 2b). Even though the sessions were cued, there still exists some variations in duration of each phase. Thus, we normalized the time according to average duration of each phase and present the data as percent of the respective cycle (Fig. 2c). Finally, the mean and standard deviation were used to represent the data (Fig. 2d).

### Heel raise phase segmentation

In order to obtain a more descriptive understanding of the heel raise, the data should be examined according to concentric, isometric and eccentric states, which are associated with rise, hold and drop phase, respectively. In this study, we employed a simple threshold on the foot pitch to segment the heel raise events. The gradient of the foot pitch was then used to further identify the rise, hold, drop phase.

### EMG signal processing

EMG signals from the PL, GM, GL and SL were obtained and processed such that the muscle recruitment patterns involved during heel raise could be analyzed. We used the envelope function of the raw EMG signals, which is common practice, by calculating the WL. It has been previously reported that the WL correlate well with muscle activation^35,36^ and can be obtained via the Eq. (1) shown below:

1 $$WL\left(t\right)=\sum_{n=t-N+2}^{t}\left|x\left(n\right)-x(n-1)\right|,$$

where $x$, $N$, and $t$ is the raw EMG signal, window size, and current sample, respectively.

### Medial lateral foot pressure distribution

In order to monitor medial and lateral bias during heel raise, we obtained the MPP from the smart insoles by calculating the medial percentage of force detected from under the metatarsal head using the equation shown below:

2 $$MPP\left(t\right)=\frac{{F}_{2}(t)}{{F}_{2}\left(t\right)+ {F}_{3}(t)} \times 100{\%},$$

where ${F}_{n}$ represents force detected from the $n$th FSR sensor and $t$ represents the current sample.

In order to compare MPP with data obtainable via Pedar-X, we obtained the ML center of pressure (COP) which is represents relative medial COP position within the width of the insole calculated using the equation shown below:

3 $$ML~ COP \left(t\right)=\frac{{W}_{pedarX} - {COP}_{x}(t)}{{W}_{pedarX}} \times 100{\%},$$

where ${W}_{pedarX}$ represents the width of the Pedar-X insole, ${COP}_{x}$ represents medial position of the COP given by Pedar-X and $t$ represents the current sample.

## Results

**Figure 3:**
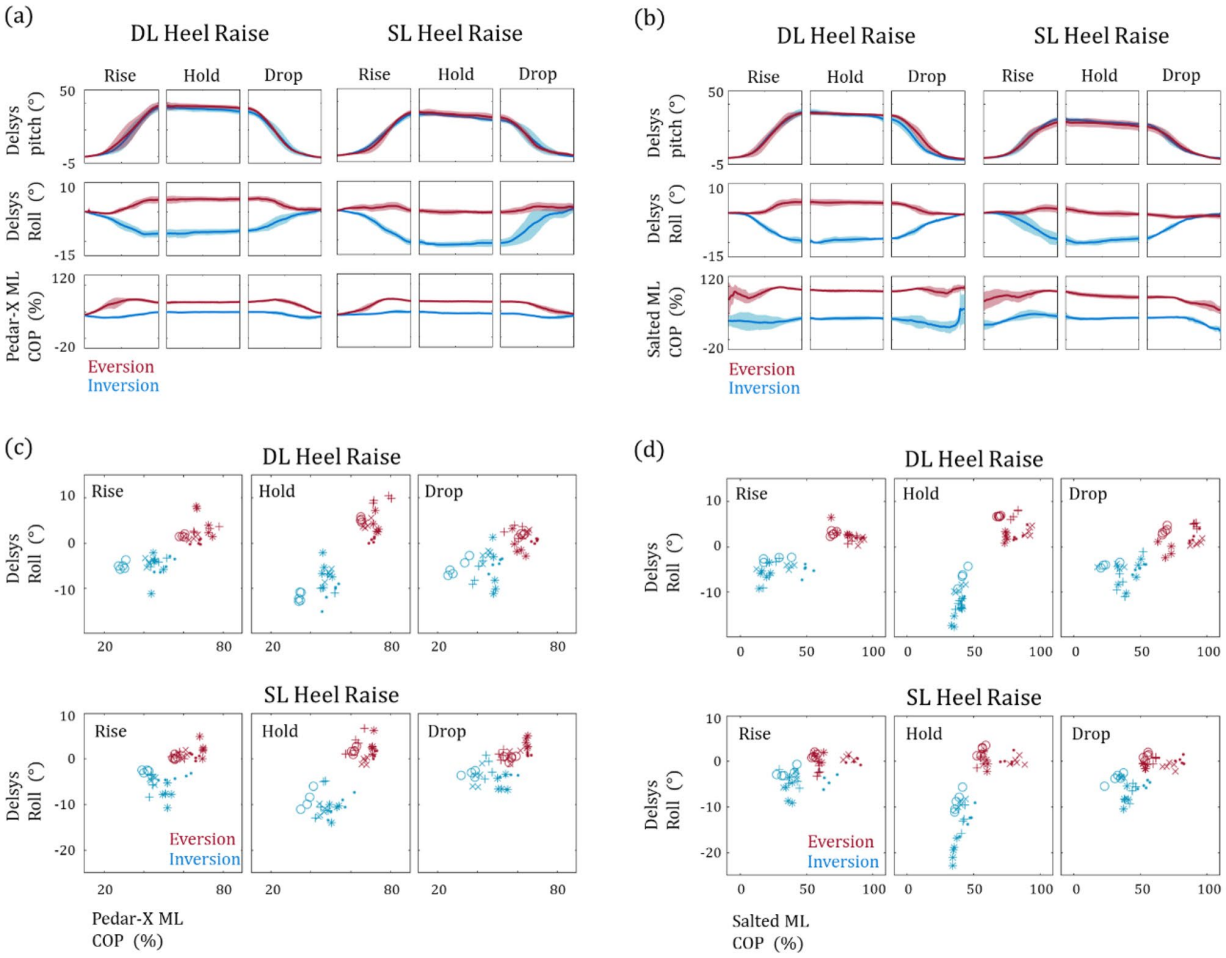
Analysis of medial–lateral foot pressure distribution during heel raise. (**a**) A representative plot of foot orientation and medial COP progression obtained via Delsys Trigno sensors and Pedar-X, respectively, during five heel raise events. The double and single leg conditions are shown on the right and left, respectively. The red lines depict heel raises with ankle eversion and the blue lines represent inversion. (**b**) Data obtained from the same subject but with the smart insoles are shown. The roll plotted against the ML COP, obtained from Pedar-X, and MPP which was obtained from the smart insoles are shown in (**c**) and (**d**), respectively. The data points represent the mean within their respective phase for each event. The double and single leg heel raise data are shown on the top and bottom panels, respectively.

Prior to any analysis with the smart insoles, we first confirmed that the four heel raise variants, DL-EV, DL-IV, SL-EV and SL-IV, were properly performed by simultaneously acquiring the foot orientation via Delsys Trigno sensors. The results clearly show an outwards rotation during the rise phase of both SL-IV and DL-IV heel raise (Fig. 3a blue lines) while an inwards or preservation of foot roll can be observed for DL-EV and SL-EV, respectively (Fig. 3a red lines). Taken together, the results suggest that the heel raise conditions were indeed properly performed.

It has been well reported that maintaining medial foot pressure during heel raise is critical when facilitating ankle eversion^19,26^. We validate this notion by examining the ML COP, acquired via Eq. (3), during the heel raise sessions (Fig. 3a bottom panel). A clear divergence in ML COP progression can be observed in respect to the ankle orientation.

Could a smart insole system which consists of only four FSR sensors still be able to discern how the heel raise was performed? To answer this question, we analyzed the MPP, calculated via Eq. (2), during the heel raise and it is clearly evident that a difference in MPP progression exists between ankle eversion and inversion during both double and single leg conditions (Fig. 3b). We note that the difference is actually more drastic than that of ML COP obtained via Pedar-X. This is most likely due to the low spatial resolution of the smart insoles rather than differences in any heel raise execution. While the ML COP represents the ML position of the current COP within the entirety of foot, MPP from the smart insoles only takes into account the foot pressure beneath the 1st and 4th metatarsal head. Also, to ensure consistency, all trials were performed in front of an orthopedic physician and the pitch and roll data further corroborate this notion because no apparent differences can be observed between the heel raises performed with Pedar-X and Salted insoles (Fig. 3a,b, top two panels).

We now investigate whether this approach is generally applicable by analyzing the roll angles against the MPP or ML COP for all five subjects (Fig. 3c,d). Each data point represents the average value within the respective phase of each heel raise event and the subjects are identified by a different marker. When comparing data points from a single individual, heel raises performed with ankle inversion tend to locate lower and more left of that obtained from heel raises performed in eversion. However, when collectively evaluated it is difficult to differentiate the two conditions solely on a single vertical line. This suggests that in order to use medial foot pressure to differentiate the two ankle conditions in real-world settings, a personalized calibration routine must be implemented.

**Figure 4:**
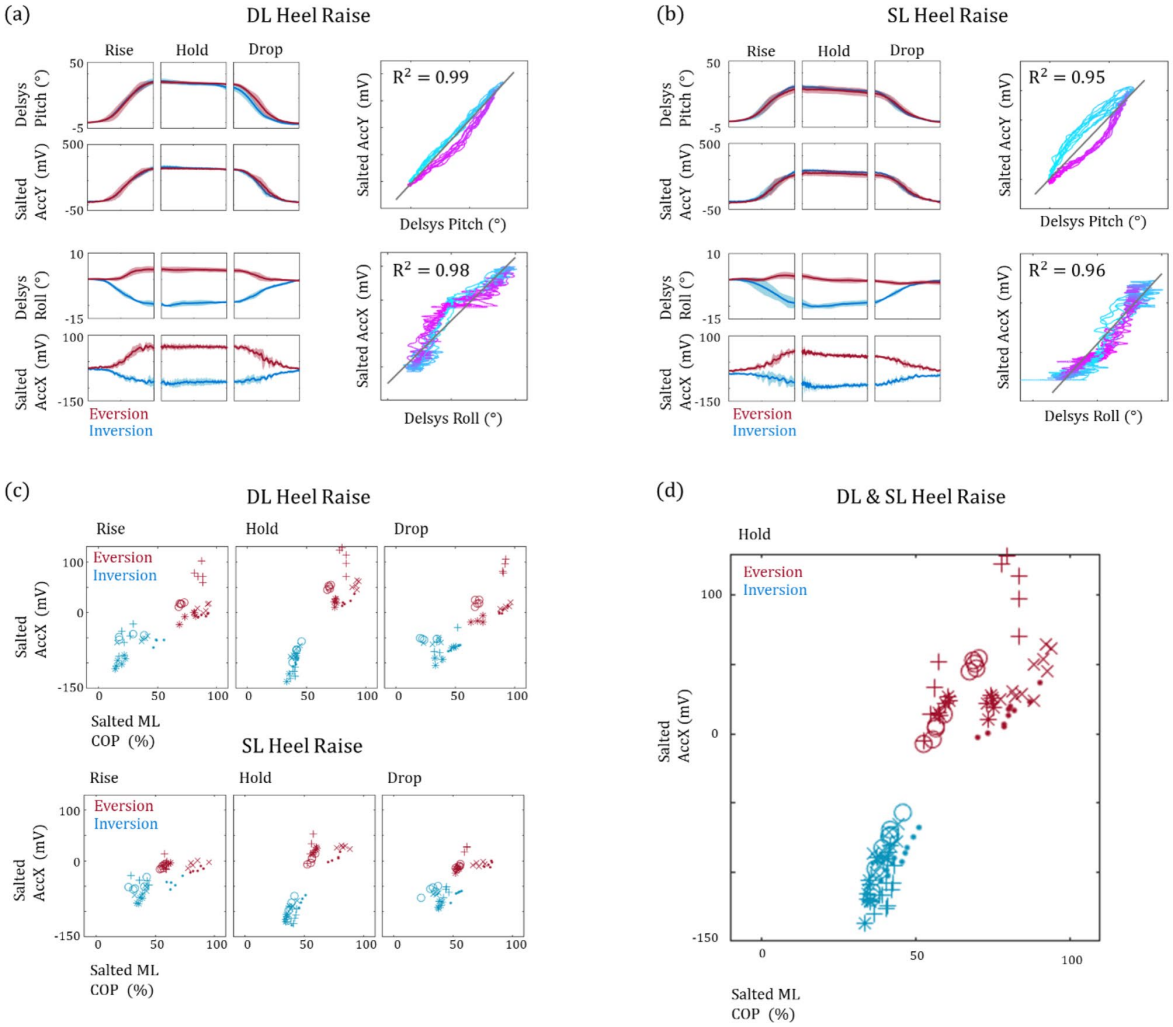
Analysis of acceleration data obtained from Salted smart insoles during heel raise. A representative plot of foot pitch and roll obtained from Delsys Trigno sensors and acceleration data from the smart insoles during (**a**) double leg and (**b**) single leg heel raise is shown. The far-right panels show their linear relationship, and the black lines represent the estimated regression line. (**c**) A distinctive separation between heel raises performed with ankle eversion and inversion from all five subjects is clearly observable on the two-dimensional plane. Both the mean roll and MPP were acquired via the smart insoles. (**d**) Data from the two ankle orientations are separable even when double and single leg conditions are not specified.

One might suggest that a possible solution to this problem could be to incorporate the data from the acceleration sensor embedded within the smart insoles because the two clusters are clearly separable on the MPP and roll plane (Fig. 3c,d). First, the feasibility of using acceleration data obtained from smart insoles to represent orientation during heel raise was confirmed by comparing y-axis and x-axis acceleration (AccY and AccX) with the pitch and roll angles acquired form Delsys Trigno sensor, respectively (Fig. 4a,b). Both values display similar trends throughout the rise, hold, and drop phases (Fig. 4a,b, left panels). Because the data from the two sensors were acquired simultaneously, we were able to evaluate the linear relationship by using a simple regression model (Fig. 4a,b, right panels). The color gradient was configured such that the initiation and termination of each heel raise event could be presented in blue and purple, respectively. We note that while a clear deviation pattern does exist, more specifically a positive and negative residual trend observed for the rise and drop phase, respectively, high ${R}^{2}$ values are observable throughout the different conditions. Since we have established that the data from the smart insoles well represent foot orientation during heel raise, we examine whether the aggregated data points from all five subjects are now separable (Fig. 4c). As postulated, with the addition of ankle orientation data the two heel raise groups are now separable on the two-dimensional plane obtained from data acquired only from the smart insoles. Furthermore, the ankle conditions without prior knowledge of single or double leg heel raise can be identified (Fig. 4d).

**Figure 5:**
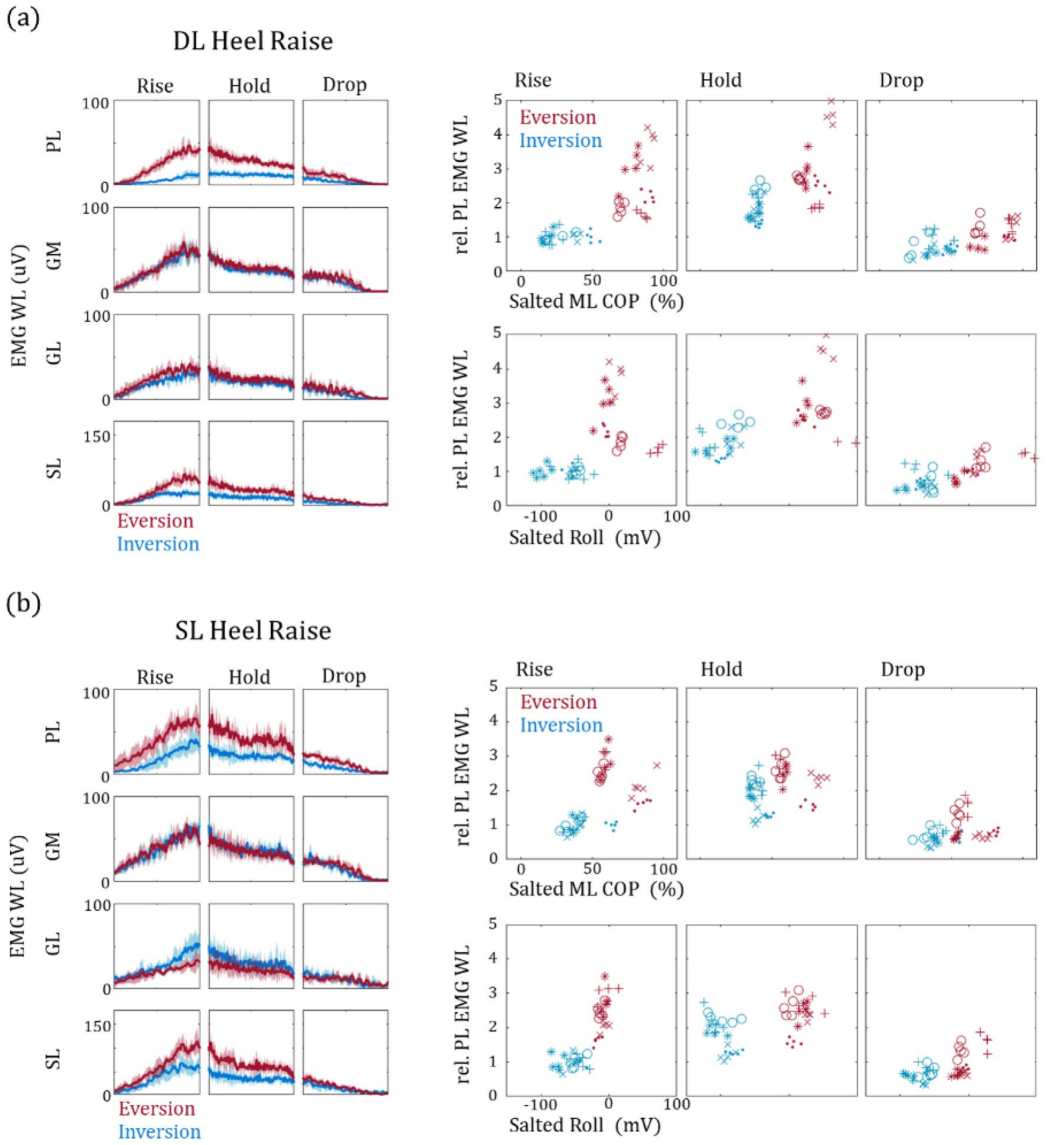
Analysis of EMG signals during heel raise. EMG data acquired from PL, GM, GL and SL during double and single leg heel raise are shown in (**a**) and (**b**), respectively. The data clearly show an increase of PL activation, during the concentric rise phase, with ankle eversion (red lines) compared to inversion (blue lines). Data from all five subjects are shown in the right panels. Because the EMG signals from different subjects cannot be directly compared, the data points are represented as relative EMG WL normalized against the mean from the respective subjects during the rise phase. The data show that ankle eversion clearly elicits an increase in PL activation during the rise phase.

We analyzed the EMG signals from PL, GM, GL and SL (Fig. 5) during the heel raise sessions. A distinct increase in PL EMG WL is observable with DL-EV and SL-EV when compared to DL-IV and SL-IV, respectively. Because the EMG signals from different subjects cannot be directly compared, we normalized the PL EMG WL to the rise phase (Fig. 5a,b, right panels). As the results show, this pattern is clearly observable for all subjects, especially during rise phase when the PL undergoes concentric contraction. Taken together, our results suggest that our smart insole system is capable of not only identifying how the heel raise was performed but more importantly assisting in the assessing PL rehabilitation.

## Discussion

In this study, we explored whether an affordable smart insole could be employed to monitor heel raise exercises and furthermore assist with CAI rehabilitation even in the absence of a clinical specialist. A total of four variants of heel raise, repeated five times, was performed by five subjects. The acceleration and foot pressure data acquired from the smart insoles were tested against Delsys Trigno sensors and Pedar-X insoles, which have been well validated. The results suggest that data acquired from Salted insoles well represent how the heel raise was performed. And with this information, we developed a simple, yet practical method to automatically detect and furthermore divide the heel raise events into rise, hold and drop phases. By dissecting and looking deeper in the data we were able to not only identify proper heel rise execution but also elucidate some intriguing findings regarding the heel raise exercise.

Firstly, while the PL EMG traces are in synchronization with GM, GL and SL during DL-EV and SL-EV, a clear delay and lack of PL activation during DL-IV and SL-IV is observed in the rise phase (Fig. 5, left panels). This is of potential interest because the EMG levels reaches its maximum during the rise phase and continuously decrease throughout the rest of the exercise even if the pitch and roll is maintained. Thus, overall PL recruitment and its potential strengthening are determined during the initial rise phase which is highly dependent on MPP (Fig. 5, right panels). Secondly, the GM and GL EMG levels are not affected by ankle orientation but change according to single or double leg conditions. This agrees well with previous studies that suggest that GM and GL activations change only when additional load is present^22,37,38^. Surprisingly, we observe that SL EMG levels changes according to ankle orientation which is in stark contrast with its fellow plantar flexors, GM and GL. This may be due to the muscle insertion position and monoarticular nature of SL, as recent studies suggest SL provide further eversion and inversion torque based on the ankle’s initial orientation^39^.

We do note that the pitch data acquired from the Delsys Trigno sensor and y-directional acceleration measured from the smart insoles may not be well modeled by a simple regression line which becomes more noticeable during SL heel raise events. We assert this to the different sensor placements, where the Delsys Trigno sensor was placed above the foot and smart insole sensor is imbedded on a level surface within the insole. The arch of the foot is known to change during loaded plantar flexion which may cause changes in partial spacing between foot and insole^4^. Most importantly, because acceleration of the foot is involved during rise and drop phase, acceleration would not well represent orientation angles. However, the two values simultaneously reach their respective maximum and minimum values which correspond with the hold phase and resting periods which allows us to accurately segment the heel raise events and phases using the smart insoles (Fig. 4, right panels).

Considering that the main objective of heel raise exercises, when prescribed for CAI patients, is to strengthen the PL muscle and that postures that exhibit increased levels of PL activation were identifiable by our system, we believe that it is now possible to conduct clinical studies including actual CAI patients. Furthermore, operation by medical professionals would not require major modifications because the smart insole data is transmitted via Bluetooth and the algorithm is simple enough to be run on any conventional smart device. Other than using acceleration and MPP to discern ankle orientation during heel raise and further ensure PL activation, various data could be extracted and utilized to assess patient rehabilitation. Possible scenarios might include tracking maximum ankle pitch and roll to assess range of motion, maximum number of DL-EV could be indicative of PL endurance and the time required to execute the rise phase could represent agility. Thus, we believe our investigation may serve as a platform to facilitate telerehabilitation, help doctors monitor at-home exercises and furthermore software as a medical device via biofeedback. We do note that our proof-of-concept study was performed with small group that lacks diversity and are currently preparing a long-term clinical study with CAI patients.
